# Prothrombin complex concentrate (Beriplex P/N) in severe bleeding: experience in a large tertiary hospital

**DOI:** 10.1186/cc6987

**Published:** 2008-08-15

**Authors:** David Bruce, Tim JC Nokes

**Affiliations:** 1 Department of Haematology, Derriford Hospital, Brest Road, Plymouth, Devon PL6 8DH, UK

## Abstract

**Introduction:**

Major blood loss can often be life-threatening and is most commonly encountered in the settings of surgery and trauma. Patients receiving anticoagulant therapy are also at increased risk of bleeding. We investigated the use of a prothrombin complex concentrate (PCC; Beriplex P/N, CSL Behring, Marburg, Germany) to treat severe bleeding in a variety of settings: cardiac surgery, warfarin therapy and other surgery.

**Methods:**

Thirty consecutive patients who had received PCC were identified from blood transfusion records. For cardiac surgery and warfarin reversal, PCC was administered in accordance with hospital protocols. PCC was administered to cardiac and other surgical patients responding poorly to recognized blood products, whereas it was administered first-line to patients with life-threatening bleeds and requiring warfarin reversal, in accordance with British Committee for Standards in Haematology guidelines. We conducted a retrospective analysis of patient records in order to ascertain PCC dose, use of other blood products and response to PCC (clotting screen results before and after PCC administration, haemostasis achievement, and survival).

**Results:**

Six patients (20%) were excluded because of inadequate documentation (*n* = 5) or acquired haemophilia (*n* = 1). Therefore, 24 patients were included in the analysis: coronary artery bypass graft (*n* = 5), mitral/aortic valve replacement (*n* = 2), other surgery (*n* = 9) and warfarin reversal (*n* = 8). Most patients (83.3%) received no more than 1500 IU of Beriplex P/N 500. Considerable reduction in administration of other blood products was seen during the 24 hours after PCC administration. Partial or complete haemostasis was achieved in 14 out of 18 cases (77.8%). In total, 12 out of 24 patients (50%) died during the study; two-thirds of the deaths were considered unrelated to bleeding. No thrombotic complications or adverse drug reactions were observed.

**Conclusion:**

This study emphasizes the value of PCC in reversing the effects of oral anticoagulant therapy in bleeding patients. It also demonstrates the potential value of PCC in controlling bleeding in patients undergoing cardiac and other surgical procedures. The use of PCC in bleeding patients without hereditary or anticoagulation-related coagulopathy is novel, and further investigation is warranted. In the future, it may be possible to use PCC as a substitute for fresh frozen plasma in this setting; adequate documentation is crucial for all blood products.

## Introduction

Major blood loss, defined as loss of 20% or more of total blood volume [1], is a significant clinical challenge and can often be life-threatening. It is most commonly encountered in surgical and trauma patients. Patients receiving anticoagulant therapy are also at increased risk of bleeding. Major bleeding contributes to approximately 30% of trauma-related deaths [1,2], starkly illustrating the need to optimize the management of affected patients.

Following immediate measures to control bleeding, the broad principles for managing massive blood loss have been summarized as follows: restore volume (administer colloids or crystalloids), perform laboratory investigations (full blood count, blood group and cross-match, coagulation screening and biochemistry), administer blood component therapy (red blood cells, platelets, fresh frozen plasma [FFP] or cryoprecipitate), and administer appropriate pharmacological agents (for instance, antifibrinolytic drugs or recombinant activated factor VII) [3]. There is therefore a variety of therapeutic options for achieving haemostasis, depending on the clinical situation.

Anticoagulant-related bleeding differs slightly in that the cause is readily identifiable as deficiency in vitamin K dependent coagulation factors. Major bleeding among patients receiving oral anticoagulant therapy (OAT) is common, affecting some 6.5% of patients per year [4]. It is often serious, with a fatality rate of approximately 1% across all age ranges, mainly due to intracerebral haemorrhage [4]. Present-day prothrombin complex concentrates (PCCs) provide a source of the four vitamin K dependent coagulation factors, and consequently these agents are recommended in both Europe and the USA for emergency anticoagulant reversal (ACR) [5-8]. Bleeding in haemophilia patients with inhibitors (for example, antibodies against factors VIII or IX) may also be treated with activated PCC [9]. Although PCC is an established therapy in these settings, little investigation of their use in bleeding related to surgery has been conducted. This could be related to historical safety concerns with PCCs [10,11], but there is considerable evidence demonstrating that thrombogenic risk has been minimized with current PCCs, especially in patients without underlying risk factors for thrombosis [12-16].

In 2001, a report of two cardiac surgery cases in which bleeding was controlled using a PCC demonstrated the potential application of PCC in this setting [17]. We were therefore keen to investigate the use of PCC to manage severe bleeding in this patient group. We were also keen to include all patients receiving PCC within our institute, partly for comparative reasons but also to provide evidence for the effectiveness of PCC in a broad patient setting. Treatment protocols were developed by consultant haematologists, for use of PCC in the settings of ACR and cardiac surgery, and we conducted a retrospective analysis of outcomes in 30 consecutive patients treated with PCC. Given this variety of cases, outcomes were considered separately for each patient group.

## Materials and methods

At Derriford Hospital in Plymouth, UK, 30 consecutive patients were identified from blood transfusion records as having received PCC (Beriplex P/N 500, CSL Behring, Marburg, Germany) over a 27-month period between April 2002 and July 2004. Each PCC vial provided factor II (400 to 960 IU), factor VII (200 to 500 IU), factor IX (400 to 620 IU) and factor X (440 to 1,200 IU), as well as the coagulation inhibitors protein C (300 to 900 IU), protein S (260 to 520 IU), antithrombin (4 to 30 IU) and heparin (8 to 40 IU). The total protein content was 6 to 14 mg/ml reconstituted solution.

For cardiac surgery, PCC was used according to a predefined hospital protocol in patients responding poorly to recognized blood products (FFP, platelets and cryoprecipitate). These more traditional blood products were administered as follows, in accordance with hospital guidelines, which were based on British Committee for Standards in Haematology guidelines [18,19]. FFP (dose 15 to 20 ml/kg) was administered when clotting was deranged and the patient was bleeding. Platelets (one adult therapeutic dose) were given when the patient's level dropped below 50 × 10^9^/l and the patient was bleeding. Cryoprecipitate (one dose equivalent to 10 packs) was administered when the fibrinogen level dropped below 1.0 g/l and the patient was bleeding. Clinical judgement was also involved in the decision-making process. Poor response, for triggering PCC administration, was defined as continued bleeding in spite of near correction of clotting parameters (activated partial thromboplastin time [APTT], prothrombin time [PT], fibrinogen level and thromboelastography; Table 1). A similar approach was adopted in patients undergoing other types of surgery (hence their receipt of PCC). In patients requiring ACR, PCC was used as first-line therapy in all patients with major bleeding, provided the thrombotic risk did not outweigh the risk of continued bleeding, in accordance with British Committee for Standards in Haematology guidelines [20].

**Table 1:** The Derriford protocols for using PCC in patients with severe bleeding associated with warfarin and cardiac reversal

Indication	Details
Cardiac surgery	There have been some limited data from a few sources regarding the benefit of using Beriplex (a PCC) within the context of life-threatening bleeding associated with cardiothoracic surgery. Much of these data in the UK are provided by the Cardiothoracic Unit of Southampton General Hospital. Investigators there have used Beriplex in more than 100 patients, both paediatric and adult, with very good effect, particularly when there are volume concerns and in the paediatric population.
	They have experienced no episodes of thrombosis when using one to two vials Beriplex P/N 500 only. 2.
	1. Beriplex should only to be given after administration of sufficient recognized blood products (FFP, platelets or cryoprecipitate) but with limited effect on bleeding in spite of documented continued coagulopathy based on laboratory data
	2. This should usually occur within the context of TEG data being unable to demonstrate clear abnormalities
	3. The maximum Beriplex usage should be two vials of P/N 500. The suggestion is to give a single vial initially, followed by a second vial if bleeding is obviously continuing. This respects the prothrombotic nature of Beriplex
	4. There should be adequate fibrinogen to produce a reasonable clot. Therefore, fibrinogen levels should be monitored and kept above 1.0 g/l if possible
	5. Note that Beriplex is held in blood transfusion and should only be administered after consultation with consultant haematologist

Anticoagulant (warfarin) reversal	In patients with life-threatening bleeding on warfarin (or other oral vitamin K antagonists), rapid reversal of anticoagulation is indicated if the thrombotic risks of complete reversal are relatively less than the risk of continuing bleeding. If the bleeding risk is greater, then Beriplex may be used for reversal on the understanding that this product is itself prothrombotic. This policy is endorsed by guidelines on oral anticoagulation [20]. Beriplex P/N 250 contains clotting factors II (200 to 480 IU), VII (100 to 250 IU), IX (200 to 310 IU) and X (220 to 600 IU), as well as protein C (150 to 450 IU) and protein S (130 to 260 IU)[13,32]. Beriplex P/N 500 contains approximately double these values of vitamin K dependent clotting factors. The 250/500 values relate to the factor IX content. In the majority of patients, FFP is not recommended because it does not completely reverse the anticoagulation effect of warfarin when it is given in quantities according to guidelines; calculated volumes would be clinically excessive [12]. FFP also requires slow thawing, which will delay reversal.
	The majority of fatal, anticoagulant-related bleeds are intracranial, in which the volume of bleeds is double [33], tend to enlarge more rapidly [34] and are associated with twice the mortality compared with intracranial bleeds in those not taking anticoagulants [33]. In order to maintain reversal, intravenous vitamin K is indicated, producing 70% correction of INR at 8 hours [33]. This dual approach is safe, with rapid replacement of clotting factors and few documented thromboses[12,13]. If possible, a repeat coagulation screen should be performed before surgery, and further doses of Beriplex should be avoided, respecting its thrombotic potential.
	There may be concerns related to the continuing anticoagulation of patients (particularly those with heart valves) after control of the bleeding episode. Advice should be sought from haematologists or cardiologists.
	**Guideline for correction of Warfarin-associated life-threatening bleeding**:
	Immediate:
	1. Beriplex P/N. Dose (INR): 25 IU/kg (2.0 to 3.9), 35 IU/kg (4.0 to 6.0) and 50 IU/kg (>6.0). For instance, eight vials of Beriplex P/N 500 for an 80 kg patient with INR >6.0. Beriplex to be given by slow intravenous injection over 10 to 15 minutes
	2. Vitamin K intravenous 2 to 5 mg
	Later:
	Consider repeating vitamin K administration 24 hours later, in those patients previously severely over-anticoagulated. An INR will help guide this decision together with clinical assessment

FFP, fresh frozen plasma; INR, International Normalized Ratio; PCC, prothrombin complex concentrate; TEG, thromboelastography.

Table 1 shows the treatment protocol implemented by the hospital for the majority of these patients. The treatment protocols, including doses, were devised from review of the literature and from the package inserts, for warfarin reversal. The time of dosing depended on clinical circumstances, as soon as major bleed suspected for warfarin reversal and upon clinical evidence of ongoing bleeding for surgical patients, together with relatively normal clotting or that which could not be corrected with traditional clotting components. In all cases, PCC was administered intravenously at a rate of approximately 15 to 20 minutes per vial.

The following details were noted for each patient: age, underlying medical condition, dose of PCC administered (quantified in IU factor IX, assuming that each vial contains 500 IU), use of other blood products (blood, FFP, cryoprecipitate and platelets), clotting screen results (before and after administration of PCC) and clinical outcomes (haemostasis and survival). Blood samples were taken before and after PCC administration for clotting screen tests (PT and APTT) and for measurement of fibrinogen levels. Although international normalized ratio (INR) is the established investigation for patients requiring control of OAT, this was frequently not recorded in this patient group. Therefore, the PT, APTT and fibrinogen results are provided wherever tested, and this provides standardization across all of the patient groups.

## Results

Of the initial 30 patients, a total of 24 were included in the analysis. Five patients were excluded because of lack of documentation of timing or administration of PCC, and one further patient with acquired haemophilia was withdrawn. The mean age of patients included in the analysis was 68.5 years (range 41 to 82 years).

Four distinct patient groups were identified: coronary artery bypass graft (CABG; *n* = 5), mitral/aortic valve replacement (*n* = 2), other surgery (*n* = 9) and warfarin reversal (*n* = 8). 'Other surgery' included an abdominal aortic aneurysm rupture repair, an infected aortic graft repair, a thoracic aorta repair after aortic valve replacement, repair of an aortic dissection, splenectomy, debulking of a gynaecological cancer and debridement of necrotizing fasciitis. Two further patients placed in this category underwent multiple procedures: one CABG and nephrectomy; and one CABG, valve replacement and abdominal aortic aneurysm repair. One of the warfarin reversal patients needed emergency surgery for bowel obstruction, and the other seven had intracranial bleeding.

The dose of PCC administered is shown in Table 2, together with the subsequent changes in blood product administration. Most patients (83.3%) received no more than 1,500 IU of PCC. Higher doses of PCC were administered to patients undergoing CABG or requiring ACR, as compared with those undergoing valve replacement or other surgery. In accordance with the protocol, the original dose of PCC for cardiac surgery was 500 to 1,000 IU only. The main reason for this restriction was the potential for thrombosis, particularly in CABG patients. However, patient records indicated that, after initial PCC treatment in accordance with the protocol, increased doses were administered if bleeding continued.

**Table 2:** Units of PCC administered per patient, and resultant change in administration of blood products

Indication	Mean dose per patient (IU [range])	Post-PCC change^a ^in administration of:			
		
		FFP	Red blood cells	Cryoprecipitate	Platelets
Cardiac surgery (CABG; *n* = 5)	1,500 (500–4,000)	-15.8%	-63.5%	-50.0%	20.0%
Cardiac surgery (valve replacement; *n* = 2)	500 (500 to 500)	-21.1%	-83.3%	-40.0%	-66.7%
Other surgery (*n* = 9)	850 (500 to 2,000)	-25.9%	-60.3%	-84.0%	-29.4%
Warfarin reversal (*n* = 8)	1,800 (1,000 to 4,000)	-50.0%	N/A	N/A	N/A
All patients (*n* = 24)	1,250 (500 to 4,000)	-22.7%	-63.9%	-70.5%	-22.2%

The dose is given as IU of factor IX, assuming that each PCC vial provides 500 IU. ^a^The percentage change was calculated by dividing the quantity of blood product administered during the 24 hours after PCC administration, by the quantity administered during the 24 hours before PCC administration; a negative value indicates a decrease after PCC administration. CABG, coronary artery bypass graft; FFP, fresh frozen plasma; N/A, not available; PCC, prothrombin complex concentrate.

**Figure 1:**
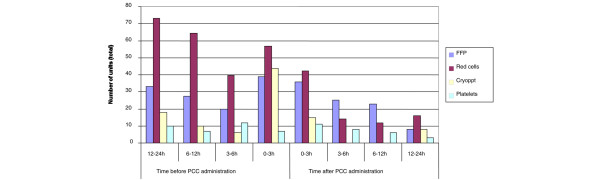
Units of blood products administered before and after PCC administration. Data shown are total numbers of units transfused for all patients (*n* = 23; data were unavailable for one patient). Cryoppt, cryoprecipitate; FFP, fresh frozen plasma; PCC, prothrombin complex concentrate.

With the exception of platelets in CABG patients, considerable reductions in the administration of all blood products were observed in all of the patient groups during the 24-hour period after PCC administration, as compared with the previous 24 hours. Figure 1 shows in greater detail the effect of PCC administration over time on patients' requirements for FFP, red blood cells, cryoprecipitate and platelets. In general terms, the reduction was not immediate; rather, it accrued over the 24-hour period after PCC administration.

For the clotting assessments, samples were taken on average 3.71 hours (range 1 to 18 hours) before PCC administration, and 5.13 hours (range 0.5 to 19 hours) after administration of PCC. The greatest percentage reduction in PT was observed in the warfarin reversal group (Table 3). For APTT, the greatest reduction was observed in the valve replacement group. Fibrinogen levels were measured in five patients before and after PCC administration. The mean pre-PCC level was 1.52 g/l, and this increased to 2.14 g/l after PCC administration – a mean increase of 40.8%. None of the patients showed a decrease in fibrinogen level after treatment with PCC.

**Table 3:** Clotting screen test results: mean PT and APTT before and after administration of PCC

Clotting parameter		Cardiac surgery (CABG)	Cardiac surgery (valve replacement)	Other surgery	Warfarin reversal	All patients
PT	Pre-PCC	19.1	24.0	32.9	52.7	35.9
	Post-PCC	15.7	13.8	15.7	14.7	15.2
	Percentage change	-17.8%	-42.5%	-52.3%	-72.1%	-57.7%
APTT	Pre-PCC	113.4	146.1	97.3	39.4	100.8
	Post-PCC	73.3	82.6	78.2	29.8	65.3
	Percentage change	-35.4%	-43.5%	-19.6%	-24.4%	-35.2%

APTT data were incomplete for one patient in the 'other surgery' group and for six patients in the warfarin reversal group, and so these patients were excluded from the analyses. APTT, activated partial thromboplastin time; CABG, coronary artery bypass graft; PCC, prothrombin complex concentrate; PT, prothrombin time.

Haemostasis outcomes were recorded in 18 patients, with partial or complete haemostasis achieved in 14 of these (77.8%; Table 4). Three out of the four patients not achieving haemostasis were in the warfarin reversal group. Seven of the 14 patients who achieved haemostasis (complete in two and partial in five) died subsequently from causes other than bleeding. In total, 12 out of 24 patients (50%) died during the study (eight [33.3%] unrelated to bleeding and four [16.6%] related to bleeding; Table 4). Although the highest death rate was observed in the valve replacement group (50%), the number of patients in this group was small and the largest number of deaths was observed among patients undergoing 'other surgery' (six fatalities among nine patients [66.7%]: four unrelated to bleeding and two related to bleeding). The warfarin reversal patient group exhibited both the highest rate of nonachievement of haemostasis and the highest survival rate; two of the three patients not achieving haemostasis survived. Two-thirds of all deaths were considered unrelated to bleeding.

**Table 4:** Clinical outcomes after PCC administration (haemostasis and survival)

Indication	Haemostasis				Survival		
		
	Complete	Partial	Not achieved	Not recorded	Death not related to bleeding	Death related to bleeding	Alive after episode
Cardiac surgery (CABG; *n* = 5)	2 (40%)	2 (40%)	0 (0%)	1 (20%)	2 (40%)	1 (20%)	2 (40%)
Cardiac surgery (valve replacement; *n* = 2)	0 (0%)	2 (100%)	0 (0%)	0 (0%)	1 (50%)	0 (0%)	1 (50%)
Other surgery (*n* = 9)	1 (11.1%)	5 (55.6%)	1 (11.1%)	2 (22.2%)	4 (44.4%)	2 (22.2%)	3 (33.3%)
Warfarin reversal (*n* = 8)	2 (25%)	0 (0%)	3 (37.5%)	3 (37.5%)	1 (12.5%)	1 (12.5%)	6 (75%)
All patients (*n* = 24)	5 (20.8%)	9 (37.5%)	4 (16.7%)	6 (25.0%)	8 (33.3%)	4 (16.6%)	12 (50%)

All values are presented as n (%). CABG, coronary artery bypass graft; PCC, prothrombin complex concentrate.

No thrombotic complications or adverse drug reactions were observed after PCC administration in any of the patients (n = 24).

## Discussion

These results demonstrate successful use of PCC in patients with severe bleeding associated with a range of circumstances, specifically cardiac and other types of surgery, and need for warfarin reversal. PCC administration was followed by a general reduction in requirement for transfusion of blood products, and haemostasis was achieved in most patients. The overall survival rate (50%) is favourable for such a group of patients with life-threatening bleeding. The majority of deaths that did occur were unrelated to bleeding.

Numerous studies have previously demonstrated the efficacy of PCCs in OAT patients requiring rapid bleeding control [13,15,21-26], and the data presented here lend further support to the already established role of PCC as preferred treatment for emergency ACR [5-8]. Coagulopathy after surgical bleeding is very different, owing to more generalized depletion of coagulation factors, and there have been no major studies of PCC in bleeding patients without pre-existing coagulopathy. One previous study [14] reported successful use of PCC in a range of critically ill patients with either active bleeding or requiring imminent surgery, but all of these patients had coagulation factor deficiencies owing to hepatic impairment. Another article [17] reported that PCC was effective in controlling bleeding in two patients undergoing cardiac surgery, but again both patients had significant liver dysfunction. There has also been a previous hospital-based audit of PCC (Beriplex P/N); all of the patients required correction of oral anticoagulation and other clotting abnormalities [27]. Again, the data showed PCC to be safe and rapidly effective. The present data are therefore novel in showing the potential application of PCC in patients undergoing cardiac surgery and other surgical procedures with severe bleeding not associated with anticoagulation therapy, haemophilia or liver disease.

It is clear that our results should be interpreted as preliminary findings only, and that more rigorous studies of PCC in the specific patient groups represented in the present cohort should be performed. The present study was retrospective (potential risk of bias is therefore accepted), and specific data on some aspects such as precise timings are unavailable. There are clear needs for optimal PCC dosing to be established and for evidence from larger patient numbers. Relative to this study, increased consistency with study methods would be valuable. Although all of the documented cardiac patients were supposed to be treated in accordance with a documented protocol, this was often violated. Greater variation was possible with warfarin patients because they were treated before the protocol had been finalised (although the same principles were applied). Moreover, future studies should include a control group. A significant limitation of the present study is that the patient outcomes cannot be attributed solely to PCC. A variety of other blood products were administered to the patients, and it is possible that – in some cases – these treatments may have produced haemostasis in the absence of PCC administration. Future studies could also include thromboelastography or thrombin generation assays, for maximum insight into the effect of PCC administration [28].

There were no thrombotic adverse events in the present study, which is important given the historical safety concerns with PCC. The lack of such adverse events is particularly comforting in the CABG patient cohort, given the administration of higher PCC doses than planned and the life-threatening nature of thrombosis in these patients.

The treatment protocol that we devised for warfarin reversal is in line with published guidelines in that setting. None of the guidelines include specific guidance in relation to PCC dosing, attributable to a lack of definitive evidence. Four studies have examined PCC dosing: one [29] indicated that 500 IU of PCC was likely to be optimal for correction of INR <5; another [30] also advocated fixed dosing, showing that 500 to 1,000 IU of PCC was adequate in most patients. Two further studies [23,31] showed that individualized dosing, based on target INR, initial INR and body weight, is an effective approach. We report successful use of individualized PCC dosing, based on initial INR and body weight, and would therefore support this approach while recognizing that further research would be valuable.

Given the lack of experience with PCCs for surgical bleeding not associated with underlying coagulopathy, it is important to consider how they might fit into the overall approach to treating such patients. The possible role of PCCs might be considered analogous to that of FFP, because both products restore levels of coagulation factors, balanced by inhibitors. We administered PCC for surgical bleeding only after inadequate response to platelets, cryoprecipitate and FFP. Perhaps the next step would be investigation of PCC as a potential substitute for FFP.

## Conclusion

The results of this study highlight a potential role for PCC in controlling bleeding in patients undergoing cardiac surgery and other surgical procedures. There is a paucity of clinical data for such broad use of PCC (including patients without underlying coagulopathy relating to OAT, haemophilia or liver dysfunction). Further investigation of bleeding in cardiac surgery and other surgical procedures would be needed to establish the optimal use of PCC in these settings.

## Key messages

• This study demonstrated successful use of PCC in patients with severe bleeding associated with cardiac and other types of surgery or need for warfarin reversal.

• Considerable reduction in the administration of other blood products was observed during the 24 hours after PCC administration.

• Partial or complete haemostasis was achieved in 14 out of 18 cases (77.8%).

• The use of PCC in bleeding patients without hereditary or anticoagulation-related coagulopathy is novel, and further investigation is warranted.

## Abbreviations

ACR: anticoagulant reversal; APTT: activated partial thromboplastin time; CABG: coronary artery bypass graft; FFP: fresh frozen plasma; INR: international normalized ratio; OAT: oral anticoagulant therapy; PCC: prothrombin complex concentrate; PT: prothrombin time.

## Competing interests

The authors declare that they have no competing interests.

## Authors' contributions

Dr Nokes played a key role in establishing the treatment protocols at Derriford Hospital and treated many of the patients included in this study. With the advice of Dr Nokes, Dr Bruce obtained the relevant patient records and extracted the data that are presented in this report. Both authors worked together with Ken Sutor in developing the manuscript.
